# Functional Bowel Complaints and the Impact on Quality of Life After Colorectal Cancer Surgery in the Elderly

**DOI:** 10.3389/fonc.2022.832377

**Published:** 2022-02-09

**Authors:** Stijn H. J. Ketelaers, Maarten van Heinsbergen, Ricardo G. Orsini, F. Jeroen Vogelaar, Joop L. M. Konsten, Grard A. P. Nieuwenhuijzen, Harm J. T. Rutten, Jacobus W. A. Burger, Johanne G. Bloemen

**Affiliations:** ^1^ Department of Surgery, Catharina Hospital, Eindhoven, Netherlands; ^2^ Department of Surgery, VieCuri Medical Centre, Venlo, Netherlands; ^3^ Department of Surgery, Elisabeth-Tweesteden Hospital, Tilburg, Netherlands; ^4^ GROW, School for Developmental Biology & Oncology, Maastricht University, Maastricht, Netherlands

**Keywords:** functional outcomes, bowel dysfunction, low anterior resection syndrome, colorectal cancer, surgery, elderly, quality of life, patient reported outcome measure (PRO)

## Abstract

**Background:**

The Low Anterior Resection Syndrome (LARS) is commonly reported after colorectal cancer surgery and significantly impairs quality of life. The prevalence and impact of LARS in the elderly after rectal cancer as well as colon cancer surgery is unclear. We aimed to describe the prevalence of LARS complaints and the impact on quality of life in the elderly after colorectal cancer surgery.

**Materials and Methods:**

Patients were included from seven Dutch hospitals if they were at least one year after they underwent colorectal cancer surgery between 2008 and 2015. Functional bowel complaints were assessed by the LARS score. Quality of life was assessed by the EORTC QLQ-C30 and EORTC QLQ-CR29 questionnaires. Outcomes in patients ≥70 years were compared to a reference group of patients <70 years.

**Results:**

In total 440 rectal cancer and 1183 colon cancer patients were eligible for analyses, of whom 133 (30.2%) rectal and 536 (45.3%) colon cancer patients were ≥70 years. Major LARS was reported by 40.6% of rectal cancer and 22.2% of colon cancer patients ≥70 years. In comparison, patients <70 years reported major LARS in 57.3% after rectal cancer surgery (p=0.001) and in 20.4% after colon cancer surgery (p=0.41). Age ≥70 years was independently associated with reduced rates of major LARS after rectal cancer surgery (OR 0.63, *p*=0.04). Patients with major LARS reported significantly impaired quality of life on almost all domains.

**Conclusion:**

Elderly should not be withheld a restorative colorectal cancer resection based on age alone. However, a substantial part of the elderly colorectal cancer patients develops major LARS after surgery, which often severely impairs quality of life. Since elderly frequently consider quality of life and functional outcomes as one of the most important outcomes after treatment, major LARS and its impact on quality of life should be incorporated in the decision-making process.

## Introduction

As a consequence of the improved treatment and outcomes in elderly colorectal cancer patients over the last decades, elderly pay more attention to long-term functional outcomes and quality of life (1, 2). In fact, elderly frequently consider outcomes related to quality of life and functional recovery at least as important as survival-related outcomes (3, 4).

The Low Anterior Resection Syndrome (LARS) is an organ-specific functional outcome that is strongly associated with quality of life (5). LARS includes a cluster of functional bowel complaints including faecal incontinence, increased stool frequency and urgency, and is prevalently observed among patients after colorectal surgery (6–8). Although LARS typically arises after rectal cancer surgery, recent studies also described these complaints after colon cancer surgery (9–11).

Functional bowel complaints after rectal cancer surgery have been described increasingly over the last decades (7, 9, 10, 12, 13). However, studies on the prevalence of LARS and the impact on quality of life among the elderly after rectal cancer as well as colon cancer surgery are scarce. It has been described earlier that faecal incontinence impairs quality of life in the elderly (14). However, the impact of LARS in the elderly, which also includes other symptoms that may impair quality of life such as urgency and increased stool frequency, is unclear. In order to adequately counsel elderly colorectal cancer patients, more knowledge is needed with regard to functional bowel complaints and the impact on quality of life in the elderly.

The primary aim of this study was to describe the prevalence of LARS complaints among elderly (≥70 years) patients after rectal cancer as well as colon cancer surgery. The secondary aim was to investigate the impact of LARS on quality of life and to compare the results in the elderly with a reference group of younger patients (<70 years).

## Materials and Methods

### Population and Data Collection

All consecutive patients that underwent colorectal cancer surgery with primary anastomosis between 2008 and 2015 in seven hospitals in The Netherlands were selected retrospectively. Patients ≥18 years who were at least 1 year after primary surgery or ostomy reversal were included. Since relapsing disease has a profound impact on quality of life, patients with metastatic or recurrent disease were excluded (15, 16). Other exclusion criteria were: presence of a (temporary) ostomy, cognitive disability or dementia, death prior to the start of the study, or a local excision, a subtotal or total colectomy or an unknown procedure.

Patient characteristics, data on treatment and pathology and additional clinical and demographic data were retrospectively extracted from the medical records. Complications occurring in the first 30 postoperative days or before hospital discharge were scored using the Clavien-Dindo classification (17). Patients received treatment according to the national treatment guidelines for colorectal cancer (18). Therefore, neoadjuvant long-course chemoradiotherapy or short-course radiotherapy was proposed in patients with stage II-III rectal cancer. Adjuvant treatment was advised in stage III colon cancer patients. Participants were approached *via* a letter that explained the aim of the study, together with the questionnaires and a prepaid return envelope. The study was reviewed and approved not to be subject to the Medical Research Involving Human Subjects Act (Medical Research Ethics Committees United – Nieuwegein, registration number W20.322).

### Measurement Instruments

The LARS score was used to assess bowel dysfunction after surgery. The LARS score is a validated questionnaire regarding functional bowel complaints (5, 19, 20). Although the LARS score was originally developed for patients after a low anterior resection, recent studies also applied the LARS score in patients after colon resections (9, 10). The questionnaire includes 5 questions with a total score ranging between 0 and 42. Based on the total score, patients are classified into: no LARS (0-20 points), minor LARS (21-29 points) or major LARS (30-42 points) (19). The European Organization for the Research and Treatment of Cancer (EORTC) QLQ-C30 and QLQ-CR29 were used to assess health-related quality of life. The EORTC QLQ-C30 questionnaire includes 30 questions on global quality of life, functional scales, and symptom assessment (21). The EORTC QLQ-CR29 includes 29 questions specifically for patients with colorectal cancer (22). For EORTC QLQ-C30 and QLQ-CR29, a high score on functional scales represents a high level of functioning and a high score on symptom scales represents a high level of symptoms.

### Statistical Analyses

The primary endpoint was the prevalence of LARS in the elderly population. Secondary endpoints were the impact of LARS complaints on quality of life, the differences in LARS outcomes between the elderly (≥70 years) and a reference group of younger patients (<70 years), and factors associated with major LARS. Statistical analyses were performed using SPSS Statistics 25.0 software (IBM, Endicott, New York, USA). Demographics were presented for all patients. Continuous data were reported as mean with standard deviation (SD) or as median with interquartile range (IQR), depending on the parameter distribution. Categorical data were reported as count with percentage (%).

Intergroup comparisons between patients ≥70 years and a reference group of patients <70 years were performed using chi-squared test or Fisher’s exact test for categorical data, when appropriate. Unpaired *t*-tests and Mann-Whitney *U* tests were used for normally and non-normally distributed continuous data. A *p*-value of <0.05 was considered statistically significant. All tests were two-sided.

The following factors possibly associated with major LARS were tested in univariable binary logistic regression analyses: gender, age at time of surgery, American Society of Anaesthesiologists (ASA) classification, time since surgery, neoadjuvant treatment (in rectal cancer), surgical procedure, surgical technique, distance of tumour from anal verge on MRI or colonoscopy in centimetres from the anal verge (in rectal cancer), temporary diverting ostomy during primary surgery, pathological tumour stage, anastomotic leakage, postoperative complications (Clavien-Dindo scale) and adjuvant chemotherapy (in colon cancer). Multivariable logistic binary regression analyses were used to test for the independent association of potential factors associated with major LARS. Only variables that were possibly associated with major LARS in univariable binary regression analysis (*p*<0.10) were included in multivariable binary regression analysis. In case the missing values of a variable in univariable and multivariable binary regression analyses exceeded 5% and were considered to be missing at random or completely at random, multiple imputation was performed to impute the missing data (23).

The EORTC Health-Related Quality of Life subdomains were scored according to the standard scoring guidelines and were compared between LARS groups (no/minor or major LARS) using Mann-Whitney *U* test. Earlier studies showed that the impact of LARS complaints on quality of life was comparable between patients with no and minor LARS as well as between patients with colon and rectal cancer (9, 24). Therefore, these groups were combined in quality of life outcomes.

## Results

In total, 5036 patients underwent colorectal cancer surgery between 2008 and 2015 in the participating hospitals. Patients were excluded due to recurrent locoregional or systemic disease (n=1159), presence of an ostomy (n=568), cognitive disability or dementia (n=71), death prior to the start of the study (n=695), local excision (n=120), subtotal or total colectomy (n=37) or unknown procedure (n=8).

**Figure 1:**
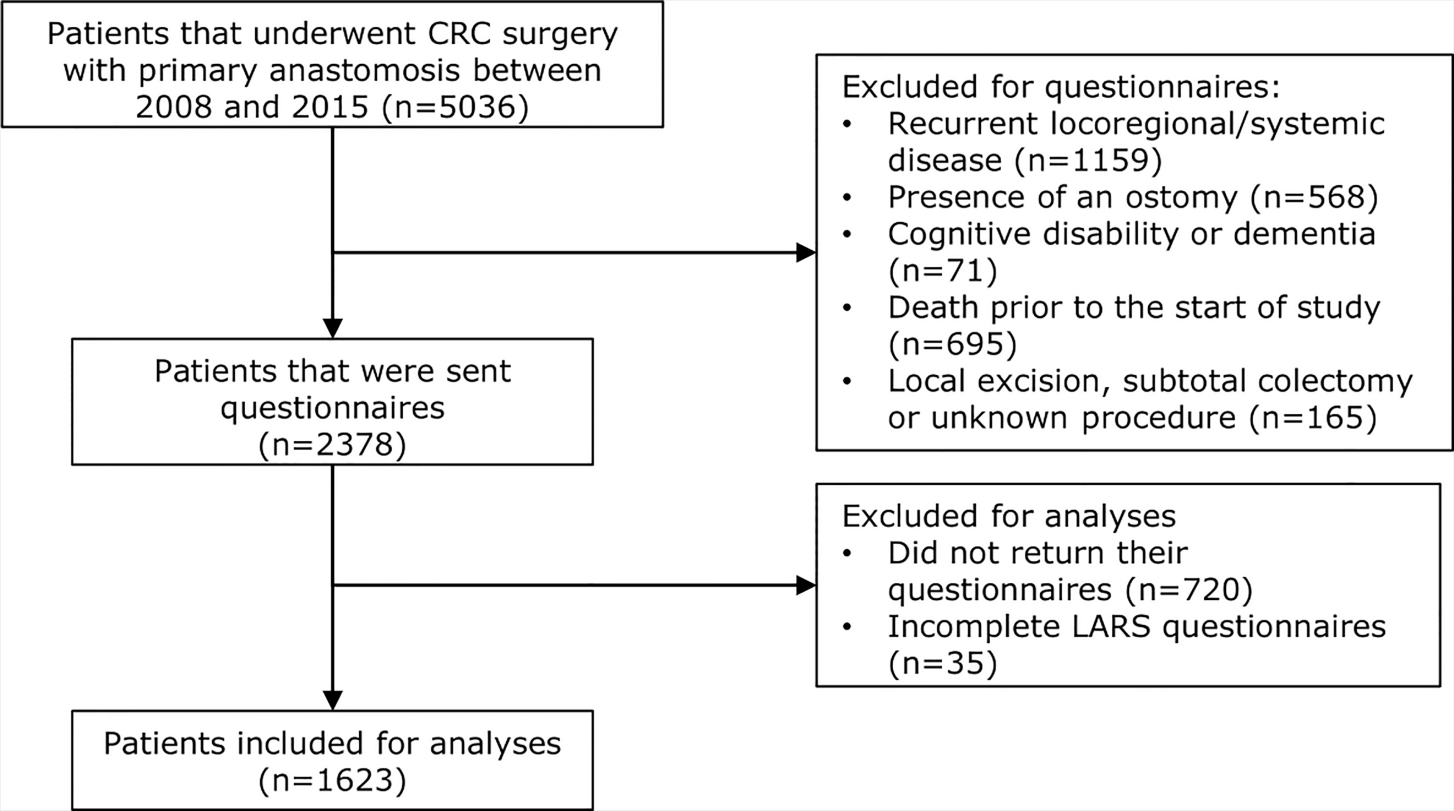
Patient disposition flowchart. Out of 5036 patients in total who underwent colorectal cancer surgery with primary anastomosis between 2008 and 2015, 2378 were sent questionnaires due to the inclusion and exclusion of patients based on the mentioned criteria. In total, 720 patients did not return their questionnaires and 35 patients returned incomplete LARS questionnaires. Overall, 1623 patients were included for analyses regarding LARS complaints and quality of life.

Eventually, 2378 patients were included in this study. A total of 1658 patients returned their EORTC QLQ-C30, EORTC QLQ-CR29 as well as their LARS questionnaires (75.6% of patients <70 years vs. 62.9% of patients ≥70, *p*<0.001). Since the primary endpoint was the prevalence of LARS, 35 patients who returned incomplete LARS questionnaires were excluded from analyses. This resulted in 1623 (68.3%) patients who were eligible for analyses. **Figure 1** presents a patient disposition flowchart.

Of the 440 rectal cancer patients, 133 (30.2%) patients were ≥70 years and 307 (69.8%) patients were <70 years. The formation of a temporary diverting ostomy during primary surgery was less often observed in patients ≥70 years than in patients <70 years (63.9% vs. 75.6%, *p*=0.01). The mean follow-up since surgery was 5.2 years (SD 2.1) for both age groups. **Table 1** presents further details on demographic outcomes in rectal cancer patients.

**Table 1:** Demographic, clinical and tumour characteristics of rectal cancer patients (n=440), stratified by age groups (<70 years and ≥70 years).

	<70 years	≥70 years	p-value
	n = 307	n = 133	
	n (%)	n (%)	
Median age in years at time of surgery (IQR)	62.2 (55.8 – 66.6)	74.5 (72.2 – 77.5)	<0.001
Mean time since surgery in years (SD)	5.2 (2.1)	5.2 (2.1)	0.94
Male	180 (58.6)	93 (69.9)	0.03
ASA classification			0.01
I-II	288 (93.8)	114 (85.7)	
≥III	13 (4.2)	16 (12.0)	
Missing	6 (2.0)	3 (2.3)	
Tumour stage			0.41
Stage 0	11 (3.6)	5 (3.8)	
Stage I-II	196 (63.8)	93 (69.9)	
Stage III-IV	100 (32.7)	35 (26.3)	
Neo-adjuvant treatment			0.01
None	97 (31.6)	47 (35.3)	
Short-course radiotherapy	96 (31.3)	57 (42.9)	
Long-course chemoradiotherapy	113 (36.8)	29 (21.8)	
Distance of tumour from anal verge (cm)			0.18
<5 cm	45 (14.7)	9 (6.8)	
5 – 9.9 cm	116 (37.8)	50 (37.6)	
10 – 14.9 cm	84 (27.4)	41 (30.8)	
≥15 cm	20 (6.5)	9 (6.8)	
Missing	42 (13.7)	24 (18.0)	
Technique of surgery			0.10
Open	164 (53.4)	68 (51.1)	
Laparoscopic	134 (43.6)	55 (41.4)	
Converted to open	8 (2.6)	10 (7.5)	
Missing	1 (0.3)	–	
Type of surgery			0.52
Rectosigmoid resection	12 (3.9)	7 (5.3)	
(Low) anterior resection	295 (96.1)	126 (94.7)	
Temporary diverting ostomy during primary surgery	232 (75.6)	85 (63.9)	0.01
Median time until ostomy reversal in months (IQR)	4.3 (2.9 – 8.0)	4.3 (3.2 – 5.6)	0.27
Anastomotic leakage	21 (6.8)	9 (6.8)	0.98
Postoperative Complications (Clavien-Dindo)			0.80
Grade 0 (No complication)	176 (57.3)	80 (60.2)	
Grade I-II	83 (27.0)	35 (26.3)	
Grade III	39 (12.7)	13 (9.8)	
Grade IV	9 (2.9)	5 (3.8)	

Of the 1183 colon cancer patients, 536 (45.3%) patients were ≥70 years and 647 (54.7%) patients were <70 years. The formation of a temporary diverting ostomy during primary surgery was less often observed in patients ≥70 years than in patients <70 years (7.3% vs. 11.9%, respectively, *p*=0.01). The mean follow-up since surgery was 4.9 years (SD 2.0) for patients ≥70 years and 4.8 years (SD 2.0) for patients <70 years (*p*=0.83). **Table 2** presents further details on demographic outcomes in colon cancer patients.

**Table 2:** Demographic, clinical and tumour characteristics of colon cancer patients (n=1183), stratified by age groups (<70 years and ≥70 years).

	<70 years	≥70 years	p-value
	n = 647	n = 536	
	n (%)	n (%)	
Median age in years at time of surgery (IQR)	63.5 (58.7 – 66.8)	76.1 (73.3 – 80.2)	<0.001
Mean time since surgery in years (SD)	4.8 (2.0)	4.9 (2.0)	0.83
Male	370 (57.2)	281 (52.4)	0.11
ASA classification			<0.001
I-II	590 (91.2)	427 (79.7)	
≥III	48 (7.4)	101 (18.8)	
Missing	9 (1.4)	8 (1.5)	
Tumour stage (pathological)			0.01
Stage 0	1 (0.2)	–	
Stage I-II	421 (65.1)	389 (72.6)	
Stage III-IV	225 (34.8)	147 (27.4)	
Technique of surgery			0.01
Open	218 (33.7)	209 (39.0)	
Laparoscopic	385 (59.5)	275 (51.3)	
Converted to open	42 (6.5)	52 (9.7)	
Missing	2 (0.3)	–	
Type of surgery			<0.001
Right hemicolectomy	251 (38.8)	276 (51.5)	
Transverse/left hemicolectomy	80 (12.4)	53 (9.9)	
Sigmoid resection	305 (47.1)	200 (37.3)	
Anterior resection	11 (1.7)	7 (1.3)	
Temporary diverting ostomy during primary surgery	77 (11.9)	39 (7.3)	0.01
Median time until ostomy reversal in months (IQR)	8.2 (4.6 – 10.1)	4.8 (3.5 – 9.4)	0.03
Anastomotic leakage	40 (6.2)	28 (5.2)	0.48
Post-operative Complications (Clavien-Dindo)			0.056
Grade 0 (No complication)	453 (70.0)	335 (62.5)	
Grade I-II	122 (18.9)	119 (22.2)	
Grade III	56 (8.7)	66 (12.3)	
Grade IV	16 (2.5)	15 (2.8)	
Missing	–	1 (0.2)	
Adjuvant chemotherapy	262 (40.5)	123 (22.9)	<0.001

### LARS Outcomes

In rectal cancer patients ≥70 years, major LARS was reported by 40.6% of patients, while minor LARS was reported by 17.9% of patients. In comparison, in patients <70 years, major LARS was reported by 57.3% (*p*=0.001) of patients and minor LARS by 19.5% (*p*=0.58) of patients.

**Figure 2:**
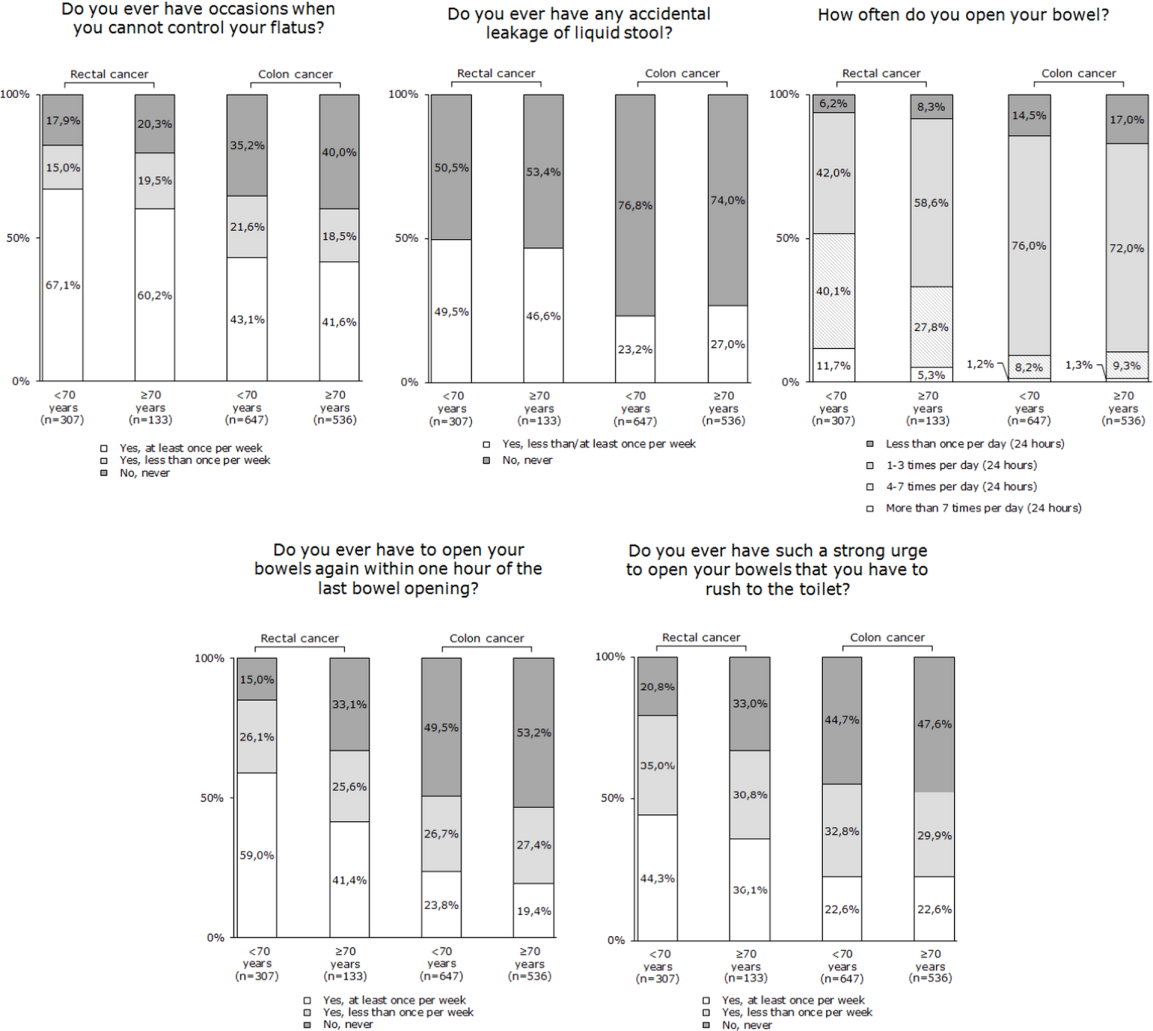
LARS subscale items, separately for rectal and colon cancer patients, stratified by age groups (<70 years and ≥70 years).

In colon cancer patients ≥70 years, major LARS was reported by 22.2% of patients and minor LARS by 17.2% of patients. In patients <70 years, major LARS was reported by 20.4% (*p*=0.41) of patients and minor LARS by 22.6% (*p*=0.02) of patients. **Figure 2** presents the subscales of the LARS score, separately for colon and rectal cancer patients.

### Associated Factors for Major LARS

Age ≥70 years was independently associated with reduced rates of major LARS in rectal cancer patients (OR 0.63, *p*=0.04). In colon cancer patients, a left hemicolectomy (OR 0.52, *p*=0.01) was independently associated with lower rates of major LARS. Female gender (OR 2.00 for rectal cancer, *p*=0.002 and OR 1.68 for colon cancer, *p*<0.001) and a temporary diverting ostomy during primary surgery (OR 2.54 for rectal cancer, *p*<0.001 and OR 1.71 for colon cancer, *p*=0.03) were independently associated with increased rates of major LARS in both rectal as well as colon cancer patients. **Tables 3** and **4** present further details of the univariable and multivariable binary regression analysis on factors associated with major LARS in rectal and colon cancer patients.

**Table 3:** Univariable and multivariable logistic regression analyses on associated factors for major LARS in rectal cancer patients (n=440).

	n (%)	Univariate			Multivariable		
		OR	95%-CI	p-value	OR	95%-CI	p-value
Gender							
Male	273 (62.0)	1.00			1.00		
Female	167 (38.0)	1.85	1.25 – 2.74	0.002	2.00	1.30 – 3.07	0.002
Age							
<70 years	307 (69.8)	1.00			1.00		
≥70 years	133 (30.2)	0.51	0.34 – 0.77	0.01	0.63	0.40 – 0.98	0.04
ASA classification							
I-II	402 (91.4)	1.00					
III	29 (6.6)	1.11	0.52 – 2.38	0.78			
Missing	9 (2.0)						
Time since surgery							
2-3 years	121 (27.5)	1.11	0.70 – 1.76	0.65			
4-5 years	134 (30.5)	1.18	0.76 – 1.85	0.46			
>5 years	185 (42.0)	1.00					
Neoadjuvant treatment							
None	144 (32.7)	1.00			1.00		
Radiotherapy	153 (34.8)	1.35	0.86 – 2.13	0.20	0.95	0.57 – 1.59	0.84
Chemoradiotherapy	142 (32.3)	2.00	1.25 – 3.21	0.004	0.94	0.53 – 1.64	0.82
Surgical procedure							
Rectosigmoid resection	19 (4.3)	1.00					
LAR	421 (95.7)	1.93	0.75 – 5.00	0.18			
Surgical technique							
Laparoscopy	189 (43.0)	1.00					
Open	232 (52.7)	1.03	0.70 – 1.51	0.88			
Converted to open	18 (4.1)	0.93	0.35 – 2.44	0.88			
Missing	1 (0.2)						
Distance of tumour from anal verge (cm)*							
<5.0 cm	60 (13.6)	4.48	2.00 – 10.07	<0.001	2.80	0.82 – 9.58	0.10
5.0-9.9 cm	183 (41.6)	2.07	1.02 – 4.18	0.04	1.41	0.64 – 3.10	0.40
10.0-14.9 cm	151 (34.3)	1.10	0.48 – 2.49	0.82	0.87	0.35 – 2.13	0.75
≥15cm	50 (11.4)	1.00			1.00		
Temporary diverting ostomy	317 (72.0)	3.10	2.00 – 4.82	<0.001	2.54	1.51 – 4.29	<0.001
Tumour stage							
Stage 0	16 (3.6)	0.82	0.29 – 2.33	0.72			
Stage I-II	289 (65.7)	0.87	0.57 – 1.30	0.49			
Stage III-IV	135 (30.7)	1.00					
Anastomotic leakage	30 (6.8)	1.21	0.57 – 2.55	0.62			
Clavien-Dindo complication grade							
Grade 0	256 (58.2)	1.00			1.00		
Grade I-II	118 (26.8)	1.22	0.79 – 1.89	0.37	1.05	0.64 – 1.71	0.85
Grade III-IV	66 (15.0)	1.99	1.13 – 3.49	0.02	1.51	0.82 – 2.80	0.19

*Multiple imputation was performed due to a high amount of missing values.

**Table 4:** Univariable and multivariable logistic regression analyses on associated factors for major LARS in colon cancer patients (n=1183).

	n (%)	Univariable			Multivariable		
		OR	95%-CI	p-value	OR	95%-CI	p-value
Gender							
Male	651 (55.0)	1.00			1.00		
Female	532 (45.0)	1.65	1.25 – 2.18	<0.001	1.68	1.26 – 2.23	<0.001
Age							
<70 years	647 (54.7)	1.00					
≥70 years	536 (45.3)	1.13	0.85 – 1.49	0.41			
ASA classification							
I-II	1017 (86.0)	1.00					
III	149 (12.6)	0.84	0.54 – 1.31	0.45			
Missing	17 (1.4)						
Time since surgery							
2-3 years	384 (32.5)	0.89	0.63 – 1.24	0.48			
4-5 years	347 (29.3)	1.15	0.82 – 1.60	0.42			
>5 years	452 (38.2)	1.00					
Surgical procedure							
Right hemicolectomy	527 (44.5)	1.00			1.00		
Left/transverse hemicolectomy	133 (11.2)	0.55	0.33 – 0.92	0.02	0.52	0.31 – 0.88	0.02
Sigmoid resection	505 (42.7)	0.77	0.57 – 1.04	0.08	0.75	0.55 – 1.03	0.07
Anterior resection	18 (1.5)	0.89	0.29 – 2.75	0.84	0.78	0.25 – 2.44	0.67
Surgical technique							
Laparoscopy	660 (55.8)	1.00					
Open	427 (36.1)	1.06	0.79 – 1.43	0.69			
Converted to open	94 (7.9)	0.82	0.47 – 1.43	0.49			
Missing	2 (0.2)						
Temporary diverting ostomy	116 (9.8)	1.54	1.00 – 2.37	0.049	1.71	1.05 – 2.79	0.03
Tumour stage							
Stage I-II	810 (68.5)	1.22	0.90 – 1.66	0.20			
Stage III-IV	372 (31.4)	1.00					
Anastomotic leakage	68 (5.7)	1.59	0.92 – 2.73	0.095	1.27	0.70 – 2.32	0.43
Clavien-Dindo complication grade							
Grade 0	788 (66.6)	1.00					
Grade I-II	241 (20.4)	1.26	0.89 – 1.77	0.20			
Grade III-IV	153 (12.9)	1.34	0.89 – 2.01	0.16			
Missing	1 (0.1)						
Adjuvant chemotherapy	385 (32.5)	0.81	0.60 – 1.10	0.17			

### Impact of LARS on Quality of Life

**Figure 3:**
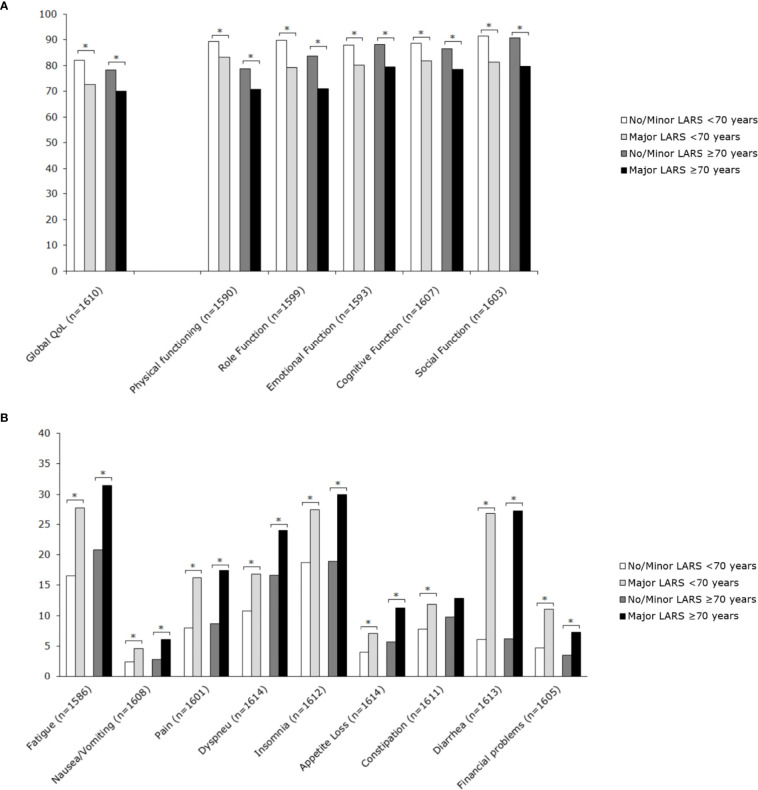
Mean scores of EORTC QLQ-C30 subscales for patients <70 years and ≥70 years, stratified by LARS group. **(A)** Functional scales: A higher score represents a better level of functioning. **(B)** Symptom scales: A higher score represents a higher level of symptoms. Significant differences are indicated by an asterisk; *p*-value < 0.05.

**Figure 4:**
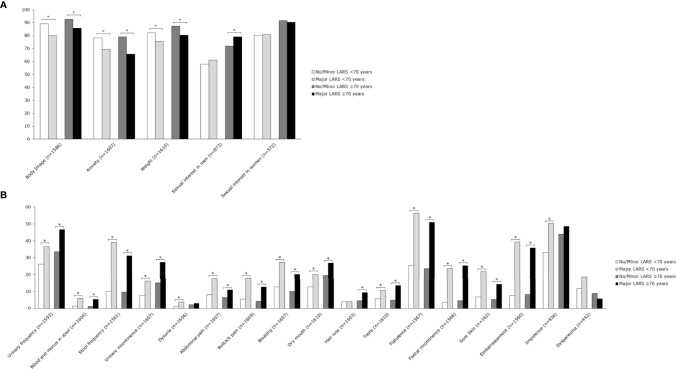
Mean scores of EORTC QLQ-CR29 subscales for patients <70 years and ≥70 years, stratified by LARS group. **(A)** Functional scales: A higher score represents a better level of functioning. **(B)** Symptom scales: A higher score represents a higher level of symptoms. Significant differences are indicated by an asterisk; *p*-value < 0.05.

Major LARS significantly impaired global quality of life in both patients ≥70 years and <70 years when compared with patients with no or minor LARS. Besides, patients with major LARS scored worse on almost all functional and symptom scales of the EORTC QLQ-C30 and EORTC QLQ-CR29 in comparison with patients with no or minor LARS. **Figures 3** and **4** present EORTC QLQ-C30 and EORTC QLQ-CR29 outcomes in both elderly and younger patients.

## Discussion

This study examined the prevalence of LARS and the impact on quality of life among elderly patients after rectal cancer as well as colon cancer surgery and addresses several important issues. Firstly, major LARS was not only prevalent in more than 40% of elderly rectal cancer patients, but also in more than 20% of elderly colon cancer patients after surgery. Secondly, our data suggest that elderly do not report major LARS more often after rectal as well as colon cancer surgery when compared with younger patients. Finally, patients that suffered from major LARS reported significantly impaired quality of life on almost all domains.

Functional bowel complaints are frequently observed after colorectal cancer surgery, especially in the first postoperative year (6, 7, 9, 10). Similar to our results, earlier studies also reported major LARS in 34-48% of patients after rectal cancer surgery and in 20% of patients after colon cancer surgery (6, 9, 11, 24). The pathophysiology of LARS after rectal as well as colon cancer surgery seems to be multifactorial (6, 25). After rectal cancer surgery, LARS is probably caused by the diminished rectal reservoir capacity, along with anal sphincter dysfunction and colonic dysmotility (6). While colonic dysmotility seems to contribute to LARS after colon cancer surgery as well, the reduced absorptive capacity of electrolytes and water probably results in more liquid stool, increased bowel frequency and the risk for faecal incontinence (9, 25–27). In accordance to the existing literature, the present study observed that LARS complaints were most prevalent in colon cancer patients after a sigmoid resection or right hemicolectomy (9, 11, 25, 27, 28). Most likely, the reduced reservoir capacity of the rectum when incorporated in an anastomosis is an additional factor contributing to these complaints after a sigmoid resection (10). While after a right hemicolectomy, the loss of the right colon, which is considered as the main site for water absorption, might contribute to the increased risk for complaints (25, 28). Besides, the loss of the ileocaecal valve and its sphincter function, the consequent ileocolic dysmotility and the increased malabsorption of bile acids in the terminal ileum may also declare the increased bowel complaints observed after a right hemicolectomy (25, 28). However, it should also be noted that among the general population without a history of abdominal surgery, 8-15% suffers from major LARS as well (7, 29). Therefore, the reported LARS rates after surgery may not be fully attributable to the treatment alone and may be pre-existent to a certain extent in some patients.

Apart from focusing on the prevalence of major LARS, it is also important to consider the impact of major LARS on quality of life. Comparing the findings of the present study with earlier studies confirms that major LARS significantly impairs quality of life on almost all domains (6, 13, 14). However, it can be hypothesized that elderly patients experience functional bowel complaints differently than younger patients. A recent study showed that elderly, especially female patients experienced less disturbances from their bowel complaints than their younger equivalents (7). A possible explanation might be that older patients more commonly suffer from a poor sphincter function, bowel complaints or faecal incontinence on forehand (14, 30). Consequently, they might be less perceptive for a deterioration of these complaints after treatment (31). Besides, elderly patients seem to adapt more rapidly to their bowel disturbances after treatment than the younger population (7). However, it must be noted that almost 50% of the elderly still considered their complaints as problematic after 2 years (7).

Clinicians often underestimate the risk and the impact of functional bowel complaints (32). However, based on the data in this study it might be suggested that in current clinical practice more attention is paid to functional bowel complaints in the elderly than in the younger population. Younger patients, which were used as a reference population in this study, reported major LARS significantly more often after rectal cancer surgery. Although some studies also found a reduced risk for major LARS in older patients, most studies did not observe any influence of age (24, 31, 33). Besides, the elderly suffer more commonly from pre-existent bowel complaints and poor sphincter function (7, 29, 30). Therefore, the reduced rates of major LARS in the elderly in this study are most likely caused by better preoperative patient selection, rather than a physiological cause (31, 33). Probably, the elderly at risk for major LARS were more often refrained from a restorative rectal cancer resection or diverting ostomy reversal when compared to younger patients (7, 24, 31, 33, 34). Since patients in whom an ostomy was still present were not included in this study, this may also clarify that temporary diverting ostomy creation was less often observed during primary surgery in the elderly when compared to the younger population. Lastly, the outcomes related to the prevalence of LARS in the elderly may also have been influenced by age-related bias, since elderly had a significantly lower response rate to the questionnaires than younger patients.

It is important for both colon and especially rectal cancer patients to be informed about major LARS and its influence on quality of life when the benefits and risks of either a restorative or non-restorative rectal cancer resection are weighed. Major LARS results in poor functional outcomes, reduced quality of life and lower levels of independency. Particularly elderly patients consider these outcomes related to functional complaints, quality of life and the maintenance of independency as one of the most important outcomes of a treatment strategy (3, 35, 36). Another aspects that should be considered in particularly in the elderly when the benefits and risk of either a restorative or non-restorative procedure are weighed, is the risk for anastomotic leakage. Especially since the elderly are at increased risk for devastating consequences if an anastomotic leakage occurs, with reported mortality rates up to 30% (37, 38). Moreover, it should be noted that a permanent end ostomy is not only well tolerated by most elderly, it also results in a quality of life that is comparable to the quality of life in the general population (39). On the contrary, there are also studies that describe an increased level of functional dependency and worse survival in rectal cancer patients with a permanent end ostomy when compared to patients with a primary anastomosis (40). Although these studies did not incorporate confounding factors that may have influenced both the decision to perform an end ostomy as well as survival, certain aspects related to an end ostomy such as the need for ostomy care, the risk for ostomy-related complications and survival should also be taken into consideration during the decision-making process.

Preoperatively discussing functional bowel complaints is crucial to set and manage expectations and to support the decision that patients have to make. The Pre-Operative Low Anterior Resection Syndrome Score (POLARS) may help as a consent aid to estimate the risk for functional bowel complaints. Apart from the value of the POLARS in counselling patients and helping them to decide between a restorative or a non-restorative resection, it may also help clinicians to identify those patients that may benefit most from a permanent end ostomy (41).

The strength of this study was the availability of real-world data from a large population of 669 elderly colorectal cancer survivors among different hospitals in The Netherlands, of whom 155 patients were older than 80 years old. Besides, we included a reference group of younger patients to put the outcomes in the elderly into perspectives. The mean time after surgery of 5 years was another strength of this study, since this has given insights in functional outcomes and the impact on quality of life on the mid- and long-term of both rectal as well as colon cancer patients.

Since data regarding patient characteristics of the excluded patients were absent and we performed a retrospective study, we did not have data regarding the preoperative patient selection, which is considered as one of our main limitations. The use of the LARS score in patients after colon resections might also be considered as a limitation, as the questionnaire was originally developed for patients after anterior resections (5). However, the application of the LARS score in colon cancer patients seems justified, since the symptoms described in the questionnaire are often reported and clinically relevant in patients after colon resections as well (9, 11, 25). This was also observed in the present study as approximately 20% of the colon cancer patients reported major LARS, which significantly impaired quality of life. A reference population of patients without previous surgery was absent, and could have given insights in the functional bowel complaints of the general elderly population. Unfortunately, we also had some missing values. Although we had to perform multiple imputation on the distance of the tumour from the anal verge in rectal cancer patients due to a high amount of missing values, most variables had no missing values at all. Despite these limitations, this study incorporated the real clinical patient selection during the included period of time. Moreover, this is the first study that reported LARS outcomes and the impact of major LARS on quality of life in a large population of elderly patients after rectal as well as colon cancer surgery. Therefore, this study provides important data that should be used in current clinical practice among elderly colorectal cancer patients. Further research is needed to better identify those elderly patients who benefit most from either an end ostomy or a primary anastomosis.

In conclusion, elderly patients should not be withheld from a restorative colorectal cancer resection based on age alone. Nevertheless, almost half of the elderly rectal cancer and one out of five elderly colon cancer patients reported major LARS after surgery, which significantly impaired quality of life. Since elderly frequently consider quality of life and functional outcomes at least as important as oncological outcomes, the risk of major LARS should be considered. When the risks and benefits of either a restorative or non-restorative procedure are weighed, counselling patients about the risk for major LARS and its impact on quality of life may be helpful.

## Data Availability Statement

The data are available upon reasonable request from the corresponding author.

## Ethics Statement

The studies involving human participants were reviewed and approved by the Medical Research Ethics Committees United – Nieuwegein. Written informed consent for participation was not required for this study in accordance with the national legislation and the institutional requirements.

## Author Contributions

SK, MH, HR, JBu, and JBl contributed to conception and design of the study. SK and MH contributed to data acquisition and database organization. SK, MH, and JBl performed the statistical analysis. All authors contributed to the interpretation of the data. SK wrote the first draft of the manuscript. All authors wrote sections of the manuscript and contributed to manuscript revision and reading. All authors approved the submitted version.

## Conflict of Interest

The authors declare that the research was conducted in the absence of any commercial or financial relationships that could be construed as a potential conflict of interest.

## Publisher’s Note

All claims expressed in this article are solely those of the authors and do not necessarily represent those of their affiliated organizations, or those of the publisher, the editors and the reviewers. Any product that may be evaluated in this article, or claim that may be made by its manufacturer, is not guaranteed or endorsed by the publisher.

## References

[1] Jansen L Koch L Brenner H Arndt V. Quality of Life Among Long-Term (≥5 Years) Colorectal Cancer Survivors - Systematic Review. Eur J Cancer (2010) 46(16):2879–88. doi: 10.1016/j.ejca.2010.06.010 20605090

[2] Caravati-Jouvenceaux A Launoy G Klein D Henry-Amar M Abeilard E Danzon A. Health-Related Quality of Life Among Long-Term Survivors of Colorectal Cancer: A Population-Based Study. Oncologist (2011) 16(11):1626–36. doi: 10.1634/theoncologist.2011-0036 PMC3233298 21987427

[3] Shrestha A Martin C Burton M Walters S Collins K Wyld L. Quality of Life Versus Length of Life Considerations in Cancer Patients: A Systematic Literature Review. Psychooncology (2019) 28(7):1367–80. doi: 10.1002/pon.5054 PMC6619389 30838697

[4] Millan M Merino S Caro A Feliu F Escuder J Francesch T. Treatment of Colorectal Cancer in the Elderly. World J Gastrointest Oncol (2015) 7(10):204–20. doi: 10.4251/wjgo.v7.i10.204 PMC4606175 26483875

[5] Emmertsen KJ Laurberg S. Low Anterior Resection Syndrome Score: Development and Validation of a Symptom-Based Scoring System for Bowel Dysfunction After Low Anterior Resection for Rectal Cancer. Ann Surg (2012) 255(5):922–8. doi: 10.1097/SLA.0b013e31824f1c21 22504191

[6] Bryant CLC Lunniss PJ Knowles CH Thaha MA Chan CLH. Anterior Resection Syndrome. Lancet Oncol (2012) 13(9):e403–8. doi: 10.1016/S1470-2045(12)70236-X 22935240

[7] Sandberg S Asplund D Bisgaard T Bock D González E Karlsson L. Low Anterior Resection Syndrome in a Scandinavian Population of Patients With Rectal Cancer: A Longitudinal Follow-Up Within the Qolirect Study. Color Dis (2020) 22(10):1367–78. doi: 10.1111/codi.15095 32346917

[8] Pieniowski EHA Nordenvall C Palmer G Johar A Tumlin Ekelund S Lagergren P. Prevalence of Low Anterior Resection Syndrome and Impact on Quality of Life After Rectal Cancer Surgery: Population-Based Study. BJS Open (2020) 4(5):935–42. doi: 10.1002/bjs5.50312 PMC7528525 32530135

[9] van Heinsbergen M den Haan N Maaskant-Braat AJ Melenhorst J Belgers EH Leijtens JW. Functional Bowel Complaints and Quality of Life After Surgery for Colon Cancer: Prevalence and Predictive Factors. Color Dis (2020) 22(2):135–45. doi: 10.1111/codi.14818 31397962

[10] van Heinsbergen M Janssen-Heijnen ML Leijtens JW Slooter GD Konsten JL. Bowel Dysfunction After Sigmoid Resection Underestimated: Multicentre Study on Quality of Life After Surgery for Carcinoma of the Rectum and Sigmoid. Eur J Surg Oncol (2018) 44(8):1261–7. doi: 10.1016/j.ejso.2018.05.003 29778617

[11] Pan LH Tsai YF. Quality of Life in Colorectal Cancer Patients With Diarrhoea After Surgery: A Longitudinal Study. J Clin Nurs (2012) 21(15–16):2357–66. doi: 10.1111/j.1365-2702.2011.04034.x 22632065

[12] Hughes DL Cornish J Morris C. Group O Behalf of the LTM. Functional Outcome Following Rectal Surgery—Predisposing Factors for Low Anterior Resection Syndrome. Int J Colorectal Dis (2017) 32(5):691–7. doi: 10.1007/s00384-017-2765-0 28130593

[13] Battersby NJ Juul T Christensen P Janjua AZ Branagan G Emmertsen KJ. Predicting the Risk of Bowel-Related Quality-of-Life Impairment After Restorative Resection for Rectal Cancer: A Multicenter Cross-Sectional Study. Dis Colon Rectum (2016) 59(4):270–80. doi: 10.1097/DCR.0000000000000552 26953985

[14] Kornmann VNN Walma MS de Roos MAJ Boerma D van Westreenen HL. Quality of Life After a Low Anterior Resection for Rectal Cancer in Elderly Patients. Ann Coloproctol (2016) 32(1):27–32. doi: 10.3393/ac.2016.32.1.27 26962533 PMC4783508

[15] Ratjen I Schafmayer C Enderle J Di Giuseppe R Waniek S Koch M. Health-Related Quality of Life in Long-Term Survivors of Colorectal Cancer and its Association With All-Cause Mortality: A German Cohort Study. BMC Cancer (2018) 18(1):1156. doi: 10.1186/s12885-018-5075-1 30466408 PMC6251222

[16] Camilleri-Brennan J Steele RJC. The Impact of Recurrent Rectal Cancer on Quality of Life. Eur J Surg Oncol (2001) 27(4):349–53. doi: 10.1053/ejso.2001.1115 11417978

[17] Dindo D Demartines N Clavien PA. Classifications of Surgical Classifications. Ann Surg (2004) 240(2):205–13. doi: 10.1097/01.sla.0000133083.54934.ae PMC1360123 15273542

[18] Federation of Medical Specialists. Dutch National Guidelines Colorectal Cancer. (2020). Available at: https://richtlijnendatabase.nl/richtlijn/colorectaal_carcinoom_crc/startpagina_-_crc.html.

[19] Juul T Ahlberg M Biondo S Emmertsen KJ Espin E Jimenez LM. International Validation of the Low Anterior Resection Syndrome Score. Ann Surg (2014) 259(4):728–34. doi: 10.1097/SLA.0b013e31828fac0b 23598379

[20] Hupkens BJP Breukink SO Olde Reuver of Briel C Tanis PJ de Noo ME van Duijvendijk P. Dutch Validation of the Low Anterior Resection Syndrome Score. Color Dis (2018) 20(10):881–7. doi: 10.1111/codi.14228 29679514

[21] Aaronson NK Ahmedzai S Bergman B Bullinger M Cull A Duez NJ. The European Organization for Research and Treatment of Cancer QLQ-C30: A Quality-of-Life Instrument for Use in International Clinical Trials in Oncology. J Natl Cancer Inst (1993) 85(5):365–76. doi: 10.1093/jnci/85.5.365 8433390

[22] Whistance RN Conroy T Chie W Costantini A Sezer O Koller M. Clinical and Psychometric Validation of the EORTC QLQ-CR29 Questionnaire Module to Assess Health-Related Quality of Life in Patients With Colorectal Cancer. Eur J Cancer (2009) 45(17):3017–26. doi: 10.1016/j.ejca.2009.08.014 19765978

[23] Jakobsen JC Gluud C Wetterslev J Winkel P. When and How Should Multiple Imputation be Used for Handling Missing Data in Randomised Clinical Trials - A Practical Guide With Flowcharts. BMC Med Res Methodol (2017) 17(1):162. doi: 10.1186/s12874-017-0442-1 29207961 PMC5717805

[24] Croese AD Lonie JM Trollope AF Vangaveti VN Ho Y-H. A Meta-Analysis of the Prevalence of Low Anterior Resection Syndrome and Systematic Review of Risk Factors. Int J Surg (2018) 56:234–41. doi: 10.1016/j.ijsu.2018.06.031 29936195

[25] Ohigashi S Hoshino Y Ohde S Onodera H. Functional Outcome, Quality of Life, and Efficacy of Probiotics in Postoperative Patients With Colorectal Cancer. Surg Today (2011) 41(9):1200–6. doi: 10.1007/s00595-010-4450-6 21874415

[26] Vather R O’Grady G Arkwright JW Rowbotham DS Cheng LK Dinning PG. Restoration of Normal Colonic Motor Patterns and Meal Responses After Distal Colorectal Resection. Br J Surg (2016) 103(4):451–61. doi: 10.1002/bjs.10074 26780492

[27] Ho YH Low D Goh HS. Bowel Function Survey After Segmental Colorectal Resections. Dis Colon Rectum (1996) 39(3):307–10. doi: 10.1007/BF02049473 8603553

[28] Hope C Reilly J Lund J Andreyev H. Systematic Review: The Effect of Right Hemicolectomy for Cancer on Postoperative Bowel Function. Supportive Care Cancer (2020) 28(10):4549–59. doi: 10.1007/s00520-020-05519-5 PMC7447648 32430603

[29] van Heinsbergen M van der Heijden JAG Stassen LP Melenhorst J de Witte E Belgers EH. The Low Anterior Resection Syndrome in a Reference Population: Prevalence and Predictive Factors in the Netherlands. Color Dis (2020) 22(1):46–52. doi: 10.1111/codi.14790 31344293

[30] Yu SWB Rao SSC. Anorectal Physiology and Pathophysiology in the Elderly. Clin Geriatr Med (2014) 30(1):95–106. doi: 10.1016/j.cger.2013.10.003 24267605 PMC3910254

[31] Bregendahl S Emmertsen KJ Lous J Laurberg S. Bowel Dysfunction After Low Anterior Resection With and Without Neoadjuvant Therapy for Rectal Cancer: A Population-Based Cross-Sectional Study. Color Dis (2013) 15(9):1130–9. doi: 10.1111/codi.12244 23581977

[32] Thomas G van Heinsbergen M van der Heijden J Slooter G Konsten J Maaskant S. Awareness and Management of Low Anterior Resection Syndrome: A Dutch National Survey Among Colorectal Surgeons and Specialized Nurses. Eur J Surg Oncol (2019) 45(2):174–9. doi: 10.1016/j.ejso.2018.11.001 30446261

[33] Sturiale A Martellucci J Zurli L Vaccaro C Brusciano L Limongelli P. Long-Term Functional Follow-Up After Anterior Rectal Resection for Cancer. Int J Colorectal Dis (2017) 32(1):83–8. doi: 10.1007/s00384-016-2659-6 27695976

[34] Hain E Manceau G Maggiori L Mongin C Prost à la Denise J Panis Y. Bowel Dysfunction After Anastomotic Leakage in Laparoscopic Sphincter-Saving Operative Intervention for Rectal Cancer: A Case-Matched Study in 46 Patients Using the Low Anterior Resection Score. Surgery (2017) 161(4):1028–39. doi: 10.1016/j.surg.2016.09.037 27894710

[35] Montroni I Ugolini G Saur NM Spinelli A Rostoft S Millan M. Personalized Management of Elderly Patients With Rectal Cancer: Expert Recommendations of the European Society of Surgical Oncology, European Society of Coloproctology, International Society of Geriatric Oncology, and American College of Surgeons Commissi. Eur J Surg Oncol (2018) 44(11):1685–702. doi: 10.1016/j.ejso.2018.08.003 30150158

[36] Orsini RG Rostoft S Rutten HJ. Rectal Cancer Treatment in the Elderly. In: Longo WE, editors. Modern Management of Cancer of the Rectum, 2nd ed. London: Springer-Verlag (2015). p. 385–403. doi: 10.1007/978-1-4471-6609-2_25

[37] Boström P Haapamäki MM Rutegård J Matthiessen P Rutegård M. Population-Based Cohort Study of the Impact on Postoperative Mortality of Anastomotic Leakage After Anterior Resection for Rectal Cancer. BJS Open (2018) 3(1):106–11. doi: 10.1002/bjs5.50106 PMC6354192 30734021

[38] Bakker IS Grossmann I Henneman D Havenga K Wiggers T. Risk Factors for Anastomotic Leakage and Leak-Related Mortality After Colonic Cancer Surgery in a Nationwide Audit. Br J Surg (2014) 101(4):424–32. doi: 10.1002/bjs.9395 24536013

[39] Orsini RG Thong MS van de P-F LV Slooter GD Nieuwenhuijzen GA Rutten HJ. Quality of Life of Older Rectal Cancer Patients is Not Impaired by a Permanent Stoma. EurJ Surg Oncol (2013) 39(2):164–70. doi: 10.1016/j.ejso.2012.10.005 23177350

[40] van Loon YT van Erning FN Maas HA Stassen LPS Zimmerman DDE. Primary Anastomosis Versus End-Ostomy in Left-Sided Colonic and Proximal Rectal Cancer Surgery in the Elderly Dutch Population: A Propensity Score Matched Analysis. Ann Surg Oncol (2021) 28(12):7450–60. doi: 10.1245/s10434-021-09976-y PMC8519826 33899138

[41] Battersby NJ Bouliotis G Emmertsen KJ Juul T Glynne-Jones R Branagan G. Development and External Validation of a Nomogram and Online Tool to Predict Bowel Dysfunction Following Restorative Rectal Cancer Resection: The POLARS Score. Gut (2018) 67(4):688–96. doi: 10.1136/gutjnl-2016-312695 28115491

